# Object Categorization Processing Differs According to Category Level: Comparing Visual Information Between the Basic and Superordinate Levels

**DOI:** 10.3389/fpsyg.2020.00501

**Published:** 2020-03-25

**Authors:** Kosuke Taniguchi, Kana Kuraguchi, Yuji Takano, Shoji Itakura

**Affiliations:** ^1^Center for Baby Science, Doshisha University, Kyoto, Japan; ^2^Faculty of Psychology, Otemon Gakuin University, Osaka, Japan; ^3^Smart-Aging Research Center, Tohoku University, Miyagi, Japan

**Keywords:** object recognition, categorization, category level, line-drawings, visual processing, word-stimulus sequence

## Abstract

Object category levels comprise a crucial concept in the field of object recognition. Specifically, categorization performance differs according to the category level of the target object. This study involved experiments with two types of stimulus sequences (i.e., forward condition: presenting the target name before the line-drawing stimulus; and reverse condition: presenting the target name after the line-drawing stimulus) for both basic- and superordinate-level categorizations. Adult participants were assigned to each level and asked to judge whether briefly presented stimuli included the same object and target name. Here, we investigated how the category level altered the categorization process. We conducted path analyses using a multivariate multiple regression model, and set our variables to investigate whether the predictors affected the categorization process between two types of stimulus sequence. Dependent variables included the measures of performance (i.e., reaction time, accuracy) for each categorization task. The predictors included dimensions and shapes of the line-drawings, such as primary and local shape information, shape complexity, subject estimation, and other shape variables related to object recognition. Results showed that the categorization process differed according to shape properties between conditions only for basic-level categorizations. For the forward condition, the bottom-up processing of primary visual information depended on matches with stored representations for the basic-level category. For the reverse condition at the basic-level category, decisions depended on subjective ratings in terms of object-representation accessibility. Finally, superordinate-level decisions depended on higher levels of visual information in terms of complexity, regardless of the condition. Thus, the given category level altered the processing of visual information for object recognition in relation to shape properties. This indicates that decision processing for object recognition is flexible depending on the criteria of the processed objects (e.g., category levels).

## Introduction

Object recognition is the foundation of various cognitive processes used in daily behavior (e.g., grabbing a cup, pointing at a target, or communicating with others). Patients with semantic dementia (a disease that affects conceptual knowledge regarding word and object meanings) may experience the deterioration of many other cognitive abilities during later stages (Rogers and Patterson, 2007). Individuals with Autism Spectrum Disorder (ASD) may also recognize objects in a different manner, thus potentially causing social difficulties (e.g., communication and behavioral deficits; Gastgeb and Strauss, 2012). To understand those who experience difficulty with object recognition, we must scrutinize the object recognition process in neurotypical adults who can judge single objects from a variety of category levels – a crucial component of object recognition. For example, most individuals who see a dog can categorize the animal as such at a glance, but can also more specifically categorize the dog (e.g., as a Siberian husky) both quickly and accurately. However, the visual information that is needed for categorization might differ between category levels (Mack and Palmeri, 2011, for psychology; Zhang et al., 2017, for computer vision). We, therefore, pose an important question: does object recognition adhere to the same process regardless of differences in the object category level? This study investigated how different category levels affected object-recognition processing performance.

Object category level is known to alter decision speed during object recognition. Rosch et al. (1976) suggested that categorization reaction time was faster when recognizing an object at an intermediate (basic; i.e., dog) level than when recognizing it at both more general (superordinate; i.e., animal) and specific (subordinate; i.e., Siberian husky) category levels. The same study indicated that basic-level categorizations were the fastest because they were conducted according to representative features in the same category. That is, individuals discriminated such features from those in other category levels.

However, basic-level categorizations are not always faster than those conducted at other levels. For example, an atypical member is categorized faster at the subordinate level (e.g., penguin) than at the basic level (e.g., bird; Jolicoeur et al., 1984). Further, a study among bird and dog experts revealed that categorizations at the subordinate level (e.g., Java sparrow) were as quick and accurate as those conducted at the basic level (e.g., bird; Tanaka and Taylor, 1991; Johnson and Mervis, 1997). On the other hand, ultra-rapid categorization tasks conducted with very brief stimuli (e.g., less than 30 ms) have shown that superordinate-level categorizations (e.g., animal) can be conducted more quickly than both basic- (e.g., dog) and subordinate-level (e.g., terrier) categorizations (Thorpe et al., 1996; Macé et al., 2009).

The representative theory of object recognition posits that visual-object processing assumes a hierarchical structure involving at least three stages. First, visual information is received by the retina, analyzed, and divided into primary information groups (e.g., edge extraction, depth segmentation, surface texture, and color). Second, an object is extracted based on this primary information. Third, the object is recognized and categorized according to a top-down matching process in which the extracted object is compared with stored representations (Rubin, 1958; Biederman, 1987; Nakayama et al., 1995; Driver and Baylis, 1996). Differences in object category levels are assumed to reflect differences in the top-down matching process completed during the third stage. Different category-level response speeds are thus the result of how object representations are accessed.

Another possible interpretation is that the required visual information differs according to the category level. As noted above, visual object recognition depends on two-way processing (i.e., bottom-up and top-down matching). Previous studies in this context have provided participants with object category levels prior to receiving the stimulus (e.g., Grill-Spector and Kanwisher, 2005). Here, given category levels may have altered bottom-up processing. As such, these studies investigated how different category levels affected the visual information needed to categorize the target object. Johnson and Olshausen (2005) indicated that forward (in which the name of a target object is given prior to the stimulus) and reverse (in which the name of a target object is given after the stimulus) conditions lead to different matching processes during object recognition. Following this, we compared the accuracy and reaction times of object recognition tasks performed with forward and reverse conditions to identify the type of processing (i.e., bottom-up or top-down) that was more effective when participants judged objects in terms of basic- or superordinate-level categories.

Various visual information types (e.g., shape, color, and texture) are used during the object recognition process, but previous research has established that shape is the most important (Marr, 1982; Biederman, 1987; Ullman, 1996). As such, this study focused on shape-related information when conducting comparisons between object category levels. Here, the curvature extrema (i.e., maximum point of curvature reached locally in a concave or convex contour) were assumed to be the most important pieces of information. Attneave (1954) showed that the most salient points were found at convex and concave points in various object contours. The curvature extrema are also crucial during the object recognition process (cf. Norman et al., 2001). Other studies have indicated that inflection points (where the curvature goes through zero locally) are also essential for object recognition (Koenderink and van Doorn, 1982; Kennedy and Domander, 1985). The present study thus investigated whether different visual information was required for the basic and superordinate levels.

**FIGURE 1:**
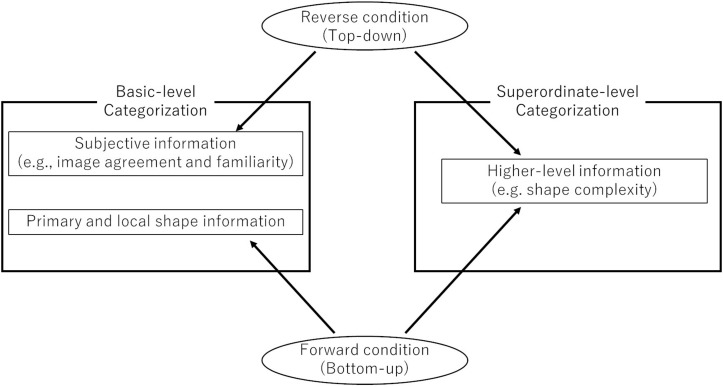
Summary of the assumption and the results of this study.

To summarize: this study specifically, (1) investigated whether decisions involving both basic- and superordinate-level categorizations depended on bottom-up and/or top-down processing according to forward and reverse conditions, and (2) specified what visual information was required at each level (Figure 1). In this study, we measured both reaction time and accuracy to specify categorization processing. This is because Taniguchi et al. (2018) showed that object recognition involves inaccurate decision with fast reaction time and accurate decision with slow reaction time. Please note that this study did not attempt to access subordinate-level categorizations through line-drawing images due to the associated difficulty (Rosch et al., 1976).

## Materials and Methods

### Participants

A total of 45 participants were selected for this study. This included 21 (eight males and 13 females aged 19–22 years) for the basic-level tasks and 24 (eight males and 16 females aged 20–28 years) for the superordinate-level tasks. All participants were Japanese undergraduate or graduate students, had normal or normal-corrected vision, and were unaware of the study’s purpose. Informed consent was obtained from all participants before conducting the experiments. Each received 1,000 yen for their participation. The Ethical Committee of the Doshisha University approved of this research (15053).

### Stimuli

**FIGURE 2:**
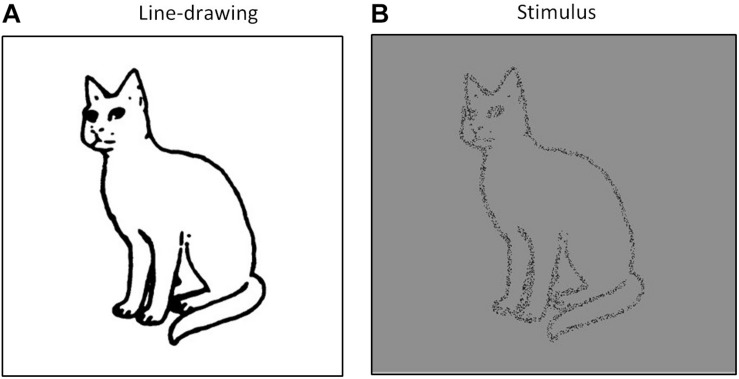
Example of a stimulus image. **(A)** Original line-drawing from Snodgrass and Vanderwart (1980) and **(B)** the stimulus image used in this study.

Line-drawing images depicting animal, plant, clothing, furniture, musical instrument, and vehicle categories were used following a study by Snodgrass and Vanderwart (1980). A total of 40 images were presented (i.e., 10 animals, 10 plants, five clothing articles, five furniture types, five musical instruments, and five vehicles). We investigated whether these images could be recognized accurately in a pilot study and showed stimulus validation with more than 88% accuracy. The stimuli were created by removing approximately 90% of the black pixels from each image (Figure 2). Here, a 50% level of black-and-white random noise was used for a mask. All stimuli were set at 8.86 × 8.86 degree of visual angles (400 × 400 pixels). All stimuli and masks were presented at the center of a 16-inch cathode-ray tube (CRT) screen (Dell E771p) over a gray background. Observation distance was set at approximately 60 centimeters, and was maintained using a chin rest. The experiments were conducted in a dark, quiet room.

### Procedure

**FIGURE 3:**
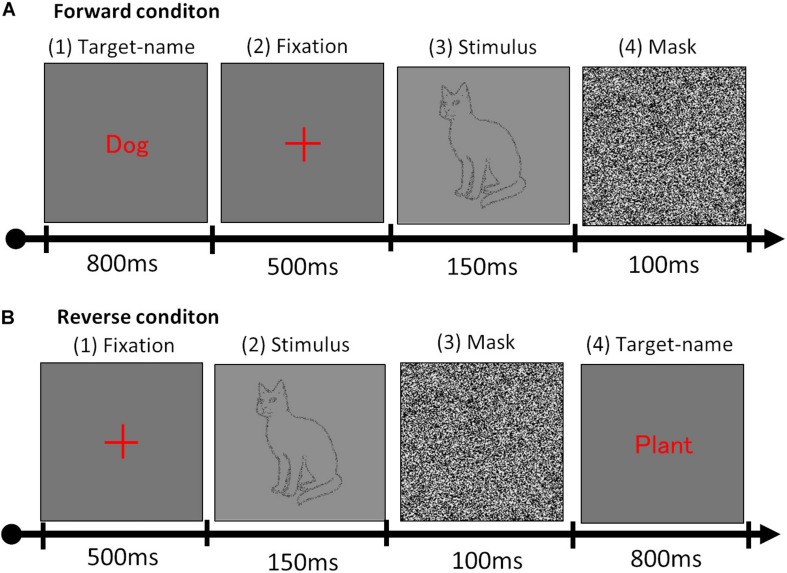
Stimulus presentation sequence. **(A)** Forward and **(B)** reverse conditions used in the experiment.

Stimuli presentation alternated between forward and reverse conditions. The forward condition presented a (1) target name for 800 ms, (2) fixation cross for 500 ms, (3) stimulus for 150 ms, and (4) mask for 100 ms (Figure 3A), while the reverse condition presented a (1) fixation cross for 500 ms, (2) stimulus for 150 ms, (3) mask for 100 ms, and (4) target name for 800 ms (Figure 3B). Target names differed according to the basic- and superordinate-level tasks. Participants were asked to announce, as soon and as accurately as possible, whether the target name was consistent with or involved in the stimulus. During basic-level tasks, there were also distractor trials in which other stimuli in the same superordinate-level categories were randomly presented (e.g., when the target name was a dog, the image of a different animal – such as a cat or elephant – was displayed). During superordinate-level tasks, other stimuli in the same natural/artifactual categories were also randomly presented (e.g., when the target was a clothing article, an object from a different category – such as a table or guitar – was displayed). Each object stimulus was presented twice for each condition (i.e., once as the target and once as the distractor). The experiments involved 80 total trials for each condition, consisting of 40 object images from six categories and congruent/incongruent trials of word-stimulus. All conditions were conducted using a blocked design and all trials were randomly assigned. Presentation and recording experiments were presented and recorded using the Windows 7 software. All participants completed eight practice trials before starting the formal experiment. The practice trials and formal tests were conducted identically, with one exception: participants were given advance knowledge of the object stimuli before starting the experiment. Each participant took approximately 30 min to complete the experiment.

### Shape Variables

This study incorporated path analyses to examine whether the shape variables involved in and estimated from the line-drawing images affected accuracy and reaction times during basic- and superordinate-level categorizations. Shape variables were calculated through image analyses or obtained from previous studies (Snodgrass and Vanderwart, 1980; De Winter and Wagemans, 2008).

The shape variables used in this study consisted of basic information from the line-drawing images, indexes of shape complexities, and subjective evaluations. The number of black pixels were counted to reflect the basic properties of each line-drawing image (Pixels). The ratio of three black pixels aligned along rows or columns in the pixel matrix was then calculated to indicate horizontal and vertical components (Matrix), while the ratio of three black pixels aligned in slanted lines to the pixels slants were calculated to indicate the slant-line components (Slant). The number of curvature singularity positions (e.g., positive maxima (M+), negative minima (m-), and inflections (I) corresponding to the positions of visual salience) was then used following De Winter and Wagemans (2008). Aspect ratio (AR) and degree of circularity (DC) were used as indices of shape complexity for the image analyses. AR refers to the ratio of horizontal and vertical contour lengths, while DC refers to the degree to which a given shape approximates circularity. Finally, subjective evaluations of visual complexity (Complexity), familiarity (Familiarity), and image agreement (ImageAgree) were used as shape variables, following Snodgrass and Vanderwart (1980).

## Results

For the trials that presented a bicycle-image stimulus, no data were collected due to a technical error. One participant was removed from each of the basic- and superordinate-level tasks and excluded from further analysis, in the first instance because mean reaction time was slower than 1,000 ms, and in the second because accuracy in the forward condition was nearly at chance level (50%). Reaction times of less than 150 ms and greater than 1,846 ms (mean [793] + 3 SD [3 × 351]) were also excluded as anticipation errors and outliers, respectively. The mean accuracy was approximately 0.93 (SD = 0.25) at basic level and 0.92 (SD = 0.27) at superordinate level. The mean reaction time was approximately 625 ms (SD = 218 ms) at basic level and 705 ms (SD = 222 ms) at superordinate level.

**FIGURE 4:**
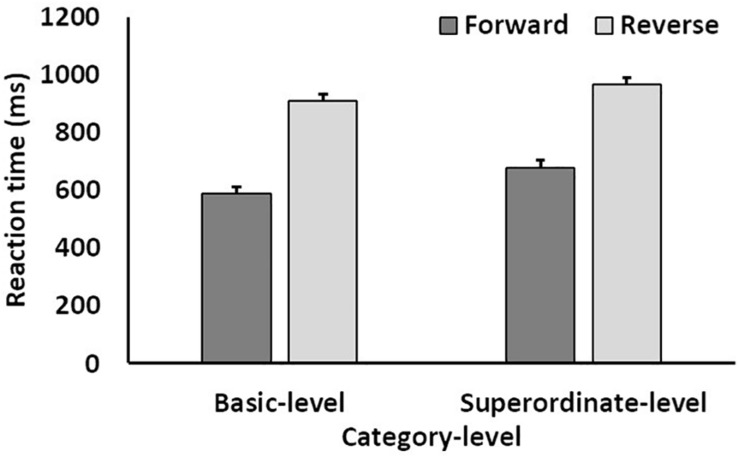
Mean reaction time as a function of target-word position (forward vs. reverse condition) and category level (basic level vs. superordinate level). Error bars indicate standard errors of the means.

A three-way mixed ANOVA was conducted for reaction time [between factor: category level; within factors: target-word position (forward vs. reverse) and stimulus category]. Results indicated that the main effects of the category level and target-word position were significant (category level and target-word position: *F*(1, 41) > 8.25, *p* < 0.01, η_p_^2^ > 0.16). The interactions between the category level and target-word position were also significant (*F*(1, 41) = 13.16, *p* < 0.001, η_p_^2^ = 0.24). The main effects of the stimulus category and other interactions were insignificant. Further, an analysis of the simple effects between the category level and target-word position showed that all single main effects were significant. This indicates that reaction times for the forward condition were shorter than those for the reverse condition at both the basic and superordinate levels, while reaction times for the basic level were shorter than those for the superordinate level for both the forward and reverse conditions (Figure 4).

**FIGURE 5:**
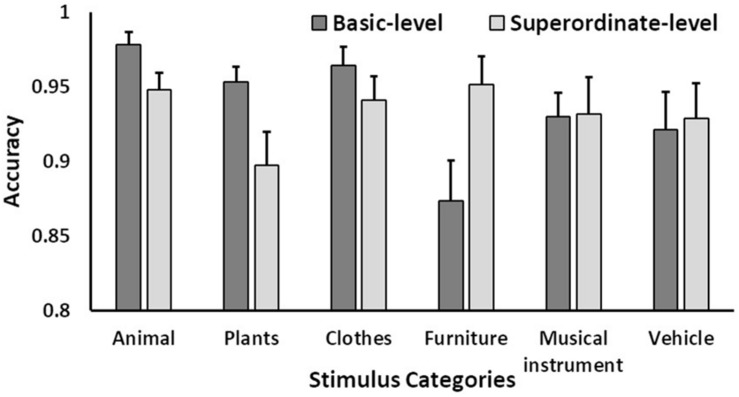
Mean accuracy as a function of category level and stimulus categories. Error bars indicate standard errors of the means.

A three-way mixed ANOVA was also conducted for accuracy (between factor: category level; within factors: target-word position and stimulus category). Results showed that the main effects of the stimulus category and interactions between category level and stimulus category were significant (stimulus category: *F*(5, 205) = 5.14, *p* < 0.01, η_p_^2^ = 0.11, ε = 0.74; category level × stimulus category: *F*(5, 205) = 7.33, *p* < 0.001, η_p_^2^ = 0.15, ε = 0.74). An analysis of the simple effect between the category level and stimulus category showed that the simple category-level effects for animals, plants, and furniture were significant (*F*(1, 41) > 6.59, *p* < 0.05, η_p_^2^ > 0.13). This indicates that accuracy was higher for the basic level than for the superordinate level for both animals and plants, whereas accuracy was higher for the superordinate level than for the basic-level for furniture. The simple main effect of the stimulus category was also significant at the basic level. Multiple comparisons indicated that the furniture category was recognized with less overall accuracy than the animal, plant, and clothing categories, while musical instruments were recognized with less accuracy than animals (Figure 5).

We then investigated the effects of visual properties on both reaction time and accuracy during categorization. This analysis was done according to a path analysis conducted with the sem package in R. The path model set reaction times for forward and reverse tasks as dependent variables, and each shape (Pixels, Matrix, Slant, M+, m−, I, AR, DC, Complexity, ImageAgree, and Familiarity) as a predictive variable. We obtained the best fitting model by estimating a saturated model containing the causal paths of all 10 predictive variables to each dependent variable (i.e., forward and reverse), correlation paths among all predictive variables (i.e., shape variables; 45 total correlations), and a correlation path between forward and reverse. Path analyses were separately conducted for the basic and superordinate levels. Paths with the highest *p*-values were deleted from the model and re-estimated for a reduced model. We conducted this reduction procedure using a backward stepwise method until all paths were significant. We then chose the lowest Akaike information criterion (AIC) model from all those estimated.

**FIGURE 6:**
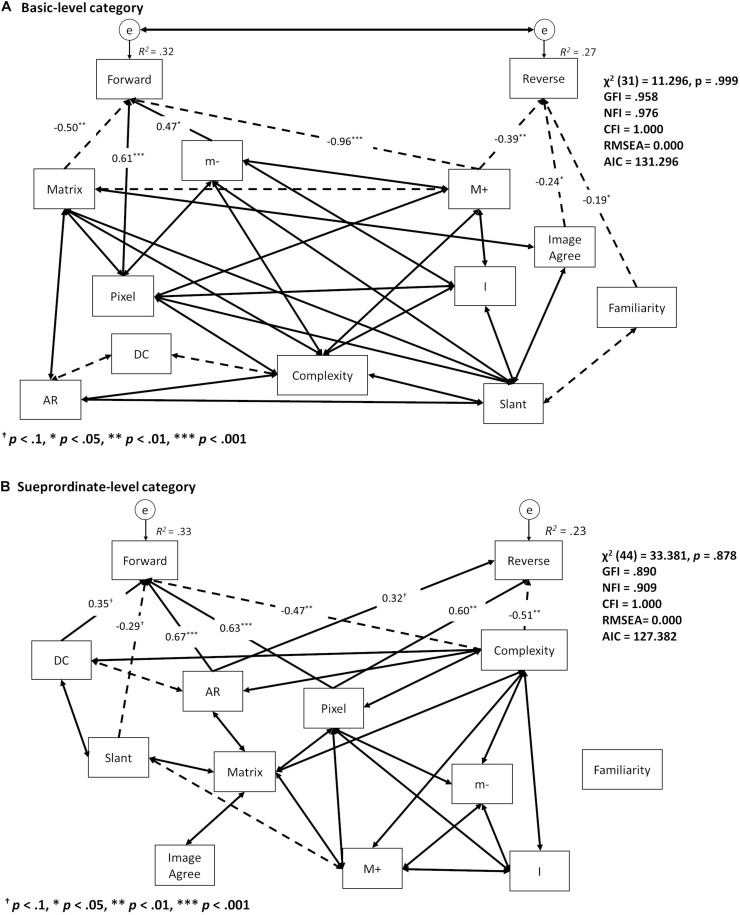
Summary of the best fitting model in the path analysis of reaction time for the **(A)** basic and **(B)** superordinate levels. One-sided arrows indicate causal effects from starting to end variables; two-sided arrows show correlations between variables. Solid arrows indicate positive effects; dashed arrows indicate negative effects. Coefficients of correlation were abbreviated for better visibility.

The best-fitting path model for reaction time at the basic level is summarized in Figure 6A (χ^2^ (31) = 11.296, *p* = 0.999, GFI = 0.958, NFI = 0.976, CFI = 1.000, RMSEA = 0.000, AIC = 131.296). Reaction time for the forward condition was significantly influenced by Pixels (β = 0.61, *p* < 0.001), Matrix (β = −0.50, *p* < 0.01), M+ (β = −0.96, *p* < 0.001), and m- (β = 0.47, *p* < 0.05), while reaction time for the reverse condition was influenced by M+ (β = −0.39, *p* < 0.01), ImageAgree (β = −0.24, *p* < 0.05), and Familiarity (β = −0.19, *p* < 0.05). Further, significant Pixels (β = 0.63, *p* < 0.001), AR (β = 0.67, *p* < 0.001) and Complexity (β = −0.47, *p* < 0.01) effects were found on reaction time for the forward condition at the superordinate level, while marginally significant effects were found for Slant (β = −0.29, *p* < 0.1) and DC (β = 0.35, *p* < 0.1). The effects of Pixels (β = 0.60, *p* < 0.01) and Complexity (β = −0.50, *p* < 0.01) were significant at the reverse condition, while effects of AR (β = 0.32, *p* < 0.1) were marginally significant (Figure 6B) (χ^2^ (44) = 33.381, *p* = 0.878, GFI = 0.890, NFI = 0.909, CFI = 1.000, RMSEA = 0.000, AIC = 127.382).

**FIGURE 7:**
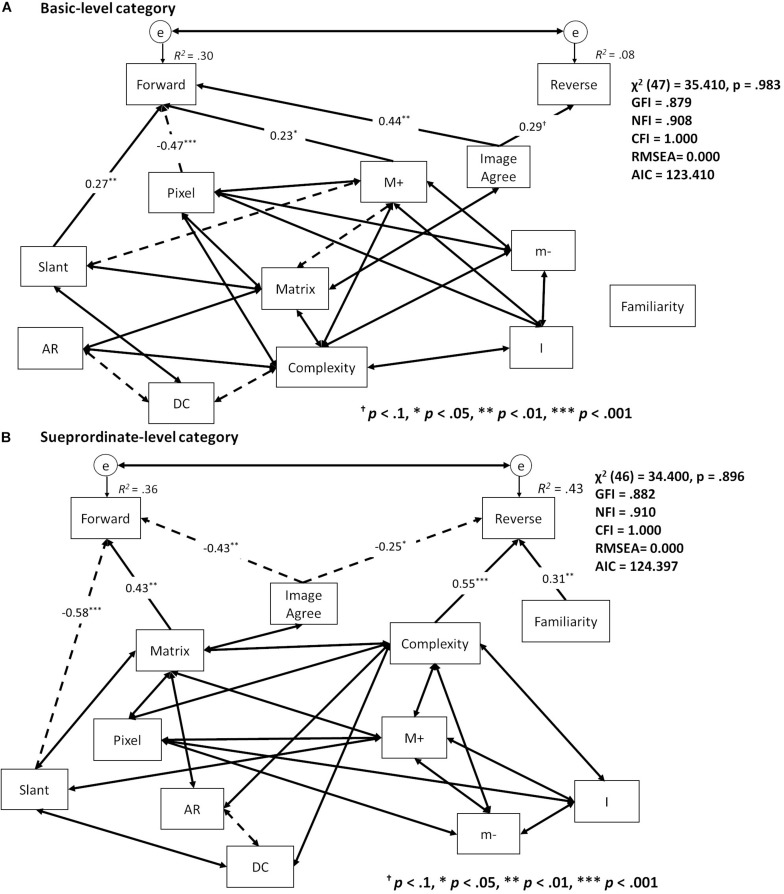
Summary of the best fitting model in the path analysis of accuracy for the **(A)** basic and **(B)** superordinate levels. One-sided arrows indicate causal effects from starting to end variables; two-sided arrows show correlations between variables. Solid arrows indicate positive effects; dashed arrows indicate negative effects. Coefficients of correlation were abbreviated for better visibility.

For the best-fitting path model for accuracy at the basic level, the effects of Pixels (β = −0.47, *p* < 0.001), Slant (β = 0.27, *p* < 0.01), M+ (β = 0.23, *p* < 0.05), and ImageAgree (β = 0.44, *p* < 0.01) were significant for the forward condition, while the effects of ImageAgree (β = 0.29, *p* < 0.1) were marginally significant for the reverse condition (Figure 7A) (χ^2^ (47) = 35.410, *p* = 0.983, GFI = 0.879, NFI = 0.908, CFI = 1.000, RMSEA = 0.000, AIC = 123.410). Accuracy for the forward condition was influenced by Matrix (β = 0.43, *p* < 0.01), Slant (β = −0.58, p < 0.001), and ImageAgree (β = −0.43, *p* < 0.001) at the superordinate level, while accuracy for the reverse condition was influenced by Complexity (β = 0.55, *p* < 0.001), Familiarity (β = 0.31, *p* < 0.01), and ImageAgree (β = −0.25, *p* < 0.05) (Figure 7B) (χ^2^ (46) = 34.400, *p* = 0.896, GFI = 0.882, NFI = 0.910, CFI = 1.000, RMSEA = 0.000, AIC = 124.397).

## Discussion

This study investigated whether bottom-up or top-down processing was preferential depending on basic- or superordinate-level categorizations. We specifically controlled the position of target cues (i.e., forward and reverse condition) in the context of a categorization task. We also investigated which shape components affected basic- and superordinate-level categorizations by comparing dependence levels in regard to shape variables. Results indicated that the categorization process changed based on both the object category levels and target-word positions. For basic-level categorizations, the most important information for the forward condition included primary and local shape properties, while that for the reverse condition included the subjective estimation of line-drawing images. For superordinate-level categorizations, however, higher-level visual information (e.g., shape complexity) was highly important regardless of whether the condition was forward or reverse.

The results of an ANOVA conducted on reaction time indicated the presence of basic-level advantage effects for both the forward and reverse conditions, thus replicating the findings of Rosch et al. (1976). For accuracy, however, such effects were only found in the animal and plant categories. On the other hand, higher accuracy was detected for the superordinate-level furniture category when compared to that of the basic level. This may indicate that accuracy in the basic level depends on the variety of objects in a category. The basic level included a greater number of items for the animal and plant categories than for others (e.g., furniture and musical instruments). For example, the animal category contained a variety of candidates (e.g., dog, cat, and bird). As such, the names of basic-level animals and plants were more familiar to participants, who exhibited higher accuracy when compared to the superordinate level. The basic-level furniture category, on the other hand, may have presented greater difficulty than other categories because constituent items contained many similar shapes (e.g., rectangles).

We also conducted a path analysis to determine the shape properties used in both the basic- and superordinate-level categorizations. Shape variables affected reaction time differently for the forward and reverse conditions only during basic-level categorizations, while the forward condition was influenced by the basic information found in line-drawings (e.g., Pixels and Matrix as well as curvature singularities such as M+ and m−). This indicated that basic-level categorizations depended on simple shape characteristics when participants matched items with stored representations during top-down processing. Further, the reverse condition was influenced by both the subjective rates of Familiarity and ImageAgree and the curvature singularities of M+. For basic-level categorizations, this may indicate that bottom-up processing is dependent on subjective ratings in terms of object-representation accessibility. Thus, shape information was prioritized differently according to “target-word position” – and, thus, mode of processing – during basic-level categorizations.

For superordinate-level categorizations, the forward and reverse conditions shared similar reaction time effects in regard to the shape variables of Pixels, AR, and Complexity. This indicates that superordinate-level categorization depends on higher-level visual information related to Complexity for both bottom-up and top-down processing.

The path analysis of accuracy indicated different dependencies on shape variables during both basic- and superordinate-level categorizations for both the forward and reverse conditions. Categorizations in the forward condition depended on basic image properties (e.g., Pixels, Matrix, and Slant), indicating that forward condition categorizations arose from shape matching and simple shape properties from stimulus images, while reverse condition categorizations arose from subjective image ratings (e.g., Complexity, Familiarity, and ImageAgree). This indicates that, for the processing of superordinate-level categorizations in the forward condition, it is more important to match simple shape properties with stored object representations, whereas for the processing of superordinate-level categorization in the reverse condition, it is more important to match the shapes stored in object representations with received visual information.

The concept of different category levels is one of the most important in object recognition. The theory of object recognition indicates that the accessibility of object representations causes different response speeds and levels of accuracy between category levels. However, this study found that such categorizations were related to different shape properties for basic and superordinate levels. We conducted experiments that were specifically designed to control the sequence of target-word positions (i.e., forward and reverse conditions), and compared visual information dependence at both levels. For basic-level categorizations, visual information differed between the forward and reverse conditions, indicating that different processes of object categorization change the required visual information through the received information. This processing difference may allow basic-level categorizations to operate in various situations both quickly and accurately. On the other hand, for superordinate-level categorizations, visual properties related to shape complexity had significant effects on reaction time and accuracy, regardless of forward or reverse conditions. As such, superordinate-level categorizations resulted in consistent responses even with minimal amounts of received information (i.e., during ultra-rapid categorizations; Thorpe et al., 1996).

In this study, we constructed a general model of object categorization and used some cross-category properties as indexes, such as a degree of circularities and curvature extrema. It is, however, possible that the indexes representing specific category properties may be involved in contribute to a more suitable model (see also, Zhang et al., 2011, 2014). Indeed, some studies have shown that categorization performance is influenced by whether an object is living or non-living (McMullen and Purdy, 2006; Riddoch et al., 2008; Mahon et al., 2009; Praß et al., 2013). Furthermore, the effect of shape properties can change based on the viewpoint of an observer (Blanz et al., 1999; Zhang et al., 2018, 2019, 2020). These issues should be investigated through further research.

Daily behavior is influenced by object recognition. This study showed that object recognition is variable, or flexible in different situations (e.g., different category levels and order of received visual information). As such, the object recognition process may change based on individual behaviors. In other words, a flexible structure was constructed during object recognition to deal with various behaviors. A previous study showed that both children and adults with ASD failed to make quick and/or accurate categorizations when compared with typically developing participants (Gastgeb et al., 2006; Gastgeb and Strauss, 2012). This may indicate that individuals with ASD have less flexibility during object recognition when compared to neurotypical individuals, thus causing difficulties in daily life. As such, additional research is necessary to clarify the relationship between object-recognition flexibility and daily behavior.

In sum, this study investigated whether categorization decisions, at both the basic and superordinate levels, depended on bottom-up and/or top-down processing according to the forward condition (presenting the target name before the line-drawing stimulus) and reverse condition (presenting the target name after the line-drawing stimulus). Further, it evaluated what visual information is required for quick and accurate categorization at each level. The results suggested that the categorization process changed based on both the object category level and target-word position. For basic-level categorizations, primary and local shape properties were important for the forward condition, while subjective estimation of line drawing images was important for the reverse condition. Superordinate-level categorizations depended on higher-level visual information (e.g., shape complexity) regardless of whether the condition was forward or reverse.

## Data Availability Statement

The raw data supporting the conclusions of this article will be made available by the authors, without undue reservation, to any qualified researcher.

## Ethics Statement

The studies involving human participants were reviewed and approved by the Ethical Committee of the Doshisha University. The patients/participants provided their written informed consent to participate in this study.

## Author Contributions

KT designed the study, conducted the experiments, and analyzed the results. KT and KK wrote the original draft of the manuscript. YT and SI supervised the project. All authors revised, read, and approved the submitted version.

## Conflict of Interest

The authors declare that the research was conducted in the absence of any commercial or financial relationships that could be construed as a potential conflict of interest.
